# Expression and prognostic value of circulating angiogenic cytokines in pancreatic cancer

**DOI:** 10.1186/1471-2407-11-286

**Published:** 2011-07-05

**Authors:** Nuh N Rahbari, Thomas Schmidt, Christine S Falk, Ulf Hinz, Magdalene Herber, Ulrich Bork, Markus W Büchler, Jürgen Weitz, Moritz Koch

**Affiliations:** 1 Department of General, Visceral and Transplant Surgery, University of Heidelberg, Heidelberg, Germany; 2 Institute for Transplant Immunology, Hannover Medical School, Hannover, Germany

## Abstract

**Background:**

The utility of circulating angiogenic cytokines (CAC) as biomarkers in pancreatic cancer has not been clarified yet. We investigated the expression and prognostic associations of seven CAC in patients with pancreatic cancer.

**Methods:**

Serum samples were collected preoperatively in patients undergoing surgery for localized pancreatic cancer (n = 74), metastatic pancreatic cancer (n = 24) or chronic pancreatitis (n = 20) and in healthy controls (n = 48). Quantitative enzyme-linked immunosorbent assays and multiplex protein arrays were used to determine circulating levels of VEGF, VEGFR-1, PlGF, PDGF-AA, PDGF-BB, Ang-1 and EGF. Multivariate analyses on cancer-specific survival were performed with a Cox proportional hazards model.

**Results:**

VEGF (p < 0.0001), PDGF-AA (p < 0.0001), Ang-1 (p = 0.002) and EGF (p < 0.0001) were differentially expressed in patients with pancreatic cancer compared to healthy controls. The presence of lymph node metastases was associated with increased levels of all CAC except for PlGF, whereas there were only minor associations of CAC with other clinicopathologic variables. The multivariate model including the entire angiogenic panel revealed high levels of circulating PDGF-AA (hazard ratio 4.58; 95% confidence interval 1.43 - 14.69) as predictor of poor cancer-specific survival, whereas high levels of PDGF-BB (0.15; 0.15 - 0.88), Ang-1 (0.30; 0.10 - 0.93) and VEGF (0.24; 0.09 - 0.57) were associated with a favorable prognosis.

**Conclusion:**

Circulating levels of certain angiogenic cytokines correlate with patients' prognosis after resection for pancreatic cancer, if a panel of several CAC is considered simultaneously. These data should be considered in future studies evaluating angiogenic factors as prognostic biomarkers and therapeutic targets in patients with pancreatic cancer.

## Background

Pancreatic cancer is ranked within the ten most common malignancies in both genders, yet it is responsible for one forth of cancer-related deaths in Western countries [1]. The poor prognosis of this disease is reflected by a dismal overall 5-year survival rate of less than 5%. Surgical resection is the only treatment modality providing a chance for cure and together with adjuvant chemotherapy may improve 5-year survival rates to 18 - 25% [2-4]. Similar to other solid malignancies the majority of patients with pancreatic cancer die of tumor progression and ultimately metastatic disease. It has become a well-established notion in tumor biology that tumor growth and progression to metastatic disease are dependent on the process of angiogenesis, i.e. the formation of new vasculature. More than 30 years ago, Folkman already postulated that adequate supply of oxygen and nutrients in tumors beyond a size of 2 - 3 mm^3 ^requires new blood vessels (i.e. perfusion), as it may not be achieved by diffusion alone [5]. The critical impact of angiogenesis for disease progression in solid tumors has been proven by data from experimental studies[6] together with the results of clinical trials that demonstrated a therapeutic effect of anti-angiogenic treatment in patients with colorectal cancer [7] and non-small-cell lung cancer [8].

The role of angiogenesis for disease progression in patients with pancreatic cancer has, however, remained less clear, as has been the potential effectiveness of anti-angiogenic therapy for this disease [9,10]. Pancreatic cancers are not grossly vascularized tumors and are rather characterized by a dense stromal reaction that might in turn promote tumor invasion [11]. Intriguingly, most pancreatic cancers display overexpression of angiogenic molecules including the vascular endothelial growth factor (VEGF) as the key mediator of tumor angiogenesis [12-14]. Nonetheless, controlled clinical trials on bevacizumab, a monoclonal antibody against VEGF and Cetuximab, a monoclonal antibody against epidermal growth factor receptor (EGFR), failed to demonstrate a survival benefit of anti-angiogenic therapy for patients with pancreatic cancer [15,16]. The failure of these agents in therapeutic trials for pancreatic cancer may in part be related to their mode of action targeting one certain molecule or its receptor. Although angiogenesis is a highly complex process that results from a misbalance of various pro- and antiangiogenic mediators [17,18], studies on the molecular biology underlying angiogenesis and the prognostic value of angiogenic cytokines in pancreatic cancer have been limited to a single or a few molecules.

Angiogenic cytokines are soluble molecules and their levels in systemic circulation may reflect the overall angiogenic activity of the tumor. Several studies could indeed demonstrate circulating angiogenic cytokines as prognostic biomarkers in patients with various solid tumors [19-22]. In the present study, we investigated the expression of CACs in patients undergoing surgery for pancreatic cancer and compared these CAC levels to the angiogenic profiles of patients with metastatic and benign pancreatic diseases. The selection of these cytokines was based on their known key roles in tumor angiogenesis[23-25]. Furthermore, we evaluated the prognostic significance of this angiogenic profile consisting of seven CACs in patients with primary pancreatic cancer.

## Methods

The primary study cohort comprised 74 patients who underwent resection for primary pancreatic cancer at the Department of General, Visceral and Transplantation Surgery, University of Heidelberg between November 2006 and April 2008. These patients had the histological diagnosis of pancreatic ductal adenocarcinoma and underwent R0 or R1 resection. Patients who received neoadjuvant chemoradiotherapy were excluded from the analysis, as were patients with a history of a second malignancy. Furthermore, we excluded patients with tumors that developed on the basis of intraductal papillary mucinous neoplasms or mucinous cystic neoplasms. To evaluate the angiogenic profile associated with metastatic disease and benign pancreatic disease, we also enrolled 24 patients who had synchronous distant metastases revealed at exploratory laparotomy and 20 patients with chronic pancreatitis who underwent surgical resection. A further control group (n = 48) included healthy subjects who had no evidence of acute or chronic disease and had no surgery within the past 12 months. All participants gave written informed consent. The study protocol was approved by the Ethics Committee of the University of Heidelberg.

Patients were treated as described previously [26]. Pathological specimens were processed using a standardized protocol [27]. R1 resection was defined, if the distance of the tumor from the resection margin was ≤ 1 mm. Adjuvant chemotherapy with gemcitabine or 5-FU was recommended to all patients who were able to tolerate it regardless of resection margin status and tumor stage. Postoperative surveillance was performed at our outpatient clinics and the European Pancreas Center (EPC). Follow-up visits were scheduled every three months in the first two years and every six months thereafter. A clinical examination, abdominal ultrasound and routine laboratory testing with evaluation of carbohydrate antigen 19-9 (CA19-9) levels were carried out at each follow-up visit. A CT scan was performed at three months postoperatively and every 6 months thereafter. To obtain follow-up information on those patients who were not followed at our institution we contacted the primary care physicians.

### Serum preparation and cytokine detection

On the day of surgery ten milliliter serum separator tubes were used to collect blood samples through a central venous catheter immediately before incision. To prevent dilution with blocking saline, the first 5 - 7 ml of the drawn blood were discarded. The blood samples were then centrifuged at 2.500 × g for 10 minutes to extract the serum; the serum was stored at -80°C until analysis. Samples of the control subjects were obtained via a peripheral vein and then processed as described above. Serum concentrations of soluble vascular endothelial growth factor receptor 1 (sVEGFR-1), placental growth factor (PlGF), platelet-derived endothelial growth factors AA (PDGF-AA), epidermal growth factor (EGF) and Angiopoetin-1 (Ang-1) were quantified using commercially available quantitative sandwich enzyme-linked immunosorbent assay (ELISA) kits (Quantikine; R&D Systems, Inc, Minneapolis, MN). All samples were analyzed in duplicate and processed at the first freeze-thaw cycle. Optical densities were quantified using a microtiter plate reader (ELISA Reader 2010, Anthos Mikrosysteme GmbH, Krefeld, Germany). The serum concentrations of vascular endothelial growth factor (VEGF) and platelet-derived endothelial growth factors BB (PDGF-BB) were determined using multiplex protein arrays (BioRad Laboratories, Hercules, CA, USA) and a two-laser array reader that simultaneously quantifies the cytokines of interest. To ensure sufficient power for subsequent survival analyses the number of included CAC was restricted to seven factors. Standard curves and concentrations were calculated using Bio-Plex Manager 4.1.1.

### Statistical analyses

Continuous data were presented as median and interquartile range and were compared using the Mann-Whitney *U *test. Categorical data were expressed as absolute and relative frequencies. A Spearman correlation coefficient ≥ 0.4 was considered to indicate a relevant correlation. Cancer-specific survival was calculated from the date of surgery for pancreatic cancer to the date of death from pancreatic cancer or the date of last follow-up information. Patients who were alive at the date of last contact were censored, as were those patients who were lost to follow-up and those who died of reasons not related to the disease. Survival curves were constructed according to the Kaplan-Meier method. The log-rank test was used for univariate comparisons of time-to-event distributions. Multivariate analyses were performed using Cox proportional hazards regression analyses. To evaluate independent prognostic associations of the individual CAC the entire panel was included in the multivariate model (i.e. regardless of their associations with survival on univariate analyses). In addition, clinicopathologic variables with a p-value < 0.2 on univariate analyses were included in the multivariate model. The levels of CAC were dichotomized using the median value. All p values were two-sided and a p-value ≤ 0.05 was considered statistically significant. All analyses were performed using SPSS^® ^software version 17 (SPSS, Chicago, Illinois, USA) and JMP program version 7 (SAS Institute Inc., Cary, NC, USA).

## Results

Table 1 summarizes the clinicopathologic characteristics of the 74 patients who underwent surgical resection for pancreatic cancer. There were 37 (50%) men and 37 women with a median age of 67.1 (58.7 - 70.7) years. The majority of patients had a tumor located in the pancreatic head (n = 54; 73%), whereas 14 (19%) and 6 (8%) patients had tumors located in the body and tail of the pancreas, respectively. Final pathological examination revealed lymph node metastases in 54 (73%) patients and microscopic margin involvement in 47 (64%) patients. Poorly differentiated tumors were diagnosed in 22 (31%) patients. A total of 49 (66%) patients received adjuvant therapy. Details on the applied adjuvant chemotherapy protocols are provided in Additional file 1.

**Table 1:** Clinicopathologic characteristics of the study population

	n (%) or median (IQR)
**Total, n**	**74 (100)**

**Gender**	
Male	37 (50.0)
Female	37 (50.0)
	
**Age [years]**	67.1 (58.7 - 70.7)
	
**Location of the tumor**	
Pancreatic head	54 (73.0)
Pancreatic body	14 (18.9)
Pancreatic tail	6 (8.1)
	
**CEA level [μg/l]**	2.9 (1.5 - 5.5)
	
**CA 19-9 level [μg/l]**	137.9 (33.8 - 558.9)
	
**Tumor size**	
T1/2	2 (2.7)
T3	72 (97.3)
	
**Lymph node status**	
Positive	54 (73.0)
Negative	20 (27.0)
	
**Resection margin status**	
R0	27 (36.5)
R1	47 (63.5)
	
**Tumor differentiation**	
Moderate (G2)	48 (68.6)
Poor (G3)	22 (31.4)
	
**Adjuvant therapy**	
Yes	49 (66.2)
No	25 (33.8)

CEA, Carcinoembryonic antigen; CA, carbohydrate antigen; IQR, Interquartile range

### Expression of circulating angiogenic cytokines in pancreatic cancer and control groups

**Figure 1:**
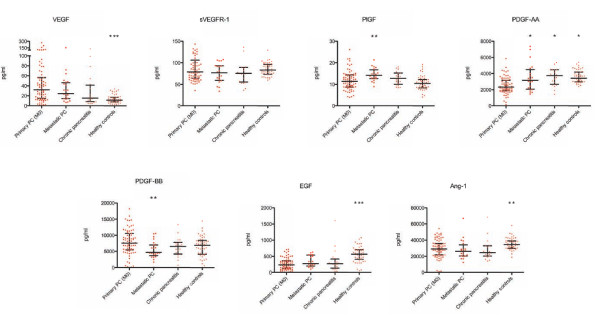
**Expression of seven circulating angiogenic cytokines in patients with primary pancreatic cancer (M0), metastatic pancreatic cancer, chronic pancreatitis and healthy control subjects**. Data are presented as median and interquartile range (black bars). * p < 0.05; ** p < 0.01; *** p < 0.001 (Mann-Whitney *U *test). Comparisons refer to the group of patients with primary pancreatic cancer.

In patients with primary pancreatic cancer, circulating levels of VEGF (p < 0.0001) were significantly increased compared to healthy control subjects, whereas circulating levels of PDGF-AA (p < 0.0001), Ang-1 (p = 0.002) and EGF (p < 0.0001) were significantly decreased. The comparison of the circulating angiogenic profile of patients with pancreatic cancer and chronic pancreatitis as benign control revealed significantly higher levels of VEGF (p = 0.05) and lower levels of PDGF-AA (p < 0.0001) in patients with malignant disease. The difference in PDGF-BB levels failed to reach statistical significance in the comparison of primary pancreatic cancer patients with healthy subjects (p = 0.07) and patients with chronic pancreatitis (p = 0.08). Certain CAC were differentially expressed in patients with primary and metastatic pancreatic cancer. While circulating levels of PlGF (p = 0.003) and PDGF-AA (p = 0.02) were significantly higher in patients with metastatic disease, these patients had lower serum concentrations of PDGF-BB (p = 0.001) (Figure 1).

### Correlation of CAC in patients with primary pancreatic cancer

The results of the correlation analyses are displayed in Table 2. There was no correlation between circulating levels of VEGFR-1 and PlGF with those of other angiogenic cytokines in patients with primary pancreatic cancer. However, we found positive correlations of PDGF-AA and Ang-1 with several CAC: PDGF-AA levels correlated with VEGF (r = 0.437), PDGF-BB (r = 0.450), Ang-1 (r = 0.755) and EGF (r = 0.429) levels. Circulating levels of Ang-1 correlated with VEGF (r = 0.401), PDGF-BB (r = 0.491) and EGF (r = 0.578).

**Table 2:** Correlation of circulating angiogenic cytokines in patients with primary pancreatic cancer

	VEGF	VEGFR-1	PlGF	PDGF-A	PDGF-B	Ang-1	EGF
**VEGF**	1	0.135	0.234	**0.437**	0.275	**0.401**	0.271
		*0.25*	*0.04*	***< 0.0001***	*0.02*	***0.0005***	*0.02*
**VEGFR-1**	0.135	1	0.164	0.201	0.270	0.292	0.066
	*0.25*		*0.16*	*0.08*	*0.02*	*0.01*	*0.57*
**PlGF**	0.234	0.164	1	0.299	0.229	0.153	0.134
	*0.04*	*0.16*		*0.01*	*0.05*	*0.19*	*0.25*
**PDGF-A**	**0.437**	0.201	0.299	1	**0.450**	**0.755**	**0.429**
	***< 0.0001***	*0.08*	*0.01*		***< 0.0001***	***< 0.0001***	***0.0001***
**PDGF-B**	0.275	0.270	0.229	**0.450**	1	**0.491**	0.206
	*0.02*	*0.02*	*0.05*	***< 0.0001***		***< 0.0001***	*0.08*
**Ang-1**	**0.401**	0.292	0.153	**0.755**	**0.491**	1	**0.578**
	***0.0005***	*0.01*	*0.19*	***< 0.0001***	***< 0.0001***		***< 0.0001***
**EGF**	0.271	0.066	0.134	**0.429**	0.206	**0.578**	1
	*0.02*	*0.57*	*0.25*	***0.0001***	*0.08*	***< 0.0001***	

The upper value indicates Spearman's correlation coefficient, whereas the lower value indicates the p-value. Relevant correlations are highlighted in bold figures.

### Association of CAC with clinicopathologic parameters in patients with primary pancreatic cancer

In a further analysis we evaluated, if circulating levels of angiogenic cytokines were associated with clinical and pathologic variables of patients with pancreatic cancer (Table 3). The results of these analyses show these associations to be rather moderate. The presence of lymph node metastases, however, correlated with increased levels of several CAC such as VEGF (p = 0.02), VEGFR-1 (p = 0.006), PDGF-AA (p = 0.04), PDGF-BB (p = 0.0008), Ang-1 (p = 0.004) and EGF (p = 0.03).

**Table 3:** Association of circulating angiogenic factors with clinicopathologic variables in patients with primary pancreatic cancer

	VEGF	VEGFR1	PlGF	PDGF-A	PDGF-B	Ang-1	EGF
**Age [years]**							
< 65	38.8 (12.6, 53.6)	86.7 (65.6, 121.9)	11.6 (9.7, 14,7)	2349.2 (1971.9, 3214.9)	7823.3 (5763.4, 10002.4)	32082.5 (27097.9, 40061.8)	261.4 (137.3, 404.4)
≥ 65	30.3 (15.8, 67.8)	76.2 (63.2, 102.8)	11.3 (8.6, 13.9)	2256.1 (1805.9, 3159.1)	7624.3 (4909.1, 11280.5)	27874.6 (20839.6, 34988.1)	229.3 (122.1, 339.6)
**Gender**							
Male	33.7 (12.6, 54.1)	76.5 (64.3, 98.5)	12.1 (9.4, 14.6)	2329.9 (1913.3, 3286.0)	7461.2 (5336.6, 11665.4)	29589.7 (23901.5, 39920.1)	276.1 (152.5, 356.3)
Female	29.8 (15.6, 59.0)	85.6 (63.3, 123.3)	11.3 (7.8, 14.1)	2342.7 (1798.8, 3162.7)	7691.8 (5627.9, 9768.8)	28157.4 (21420.4, 34759.1)	185.1 (102.3, 373.2)
**CA 19-9 level [μg/l]**							
< 37	29.2 (13.9, 47.9)	76.6 (68.7, 99.2)	10.0 (7.4, 14.8)	2224.5 (1889.7, 3077.1)	7685.5 (5662.1, 9890.9)	25991.6 (20353.1, 31308.6)	197.7 (92.6, 283.8)
≥ 37	36.9 (13.9, 73.3)	79.0 (63.3, 114.2)	12.1 (9.3, 14.3)	2342.7 (1840.9, 3342.9)	7556.8 (4851.8, 10790.6)	30517.1 (22698.9, 39237.5)	283.4 (130.6, 400.7)
**Lymph node status**							
Positive	39.6 (17.4, 68.7)*	**84.3 (68.9, 120.7)**^**§**^	12.1 (9.3, 14.4)	2370.2 (1888.7, 3417.9)*	**8658.4 (6787.3, 11406.9)**^**#**^	**30907.3 (25670.3, 41267.9)**^**§**^	286.1 (146.8, 391.3)*
negative	**21.3 (8.3, 40.7)**	**67.2 (56.9, 85.1)**	10.2 (8.2, 14.3)	**2096.8 (1795.8, 2623.6)**	**5627.9 (4566.7, 7215.9)**	**24700.5 (16330.9, 31600.9)**	**171.6 (84.1, 253.2)**
**Tumor differentiation**							
Moderate (G2)	31.6 (12.8, 55.3)	78.8 (63.1, 119.7)	10.6 (8.5, 13.7)	2336.3 (1869.7, 2850.1)	7693.9 (5448.5, 10587.1)	28568.6 (21839.1, 34451.8)	224.8 (133.4, 333.3)
Poor (G3)	39.0 (21.2, 66.7)	80.4 (67.7, 100.6)	12.6 (10.1, 15.8)	2561.1 (1853.7, 3491.3)	7756.3 (4608.0, 11545.1)	33120.3 (22580.3, 40538.4)	282.5 (112.2, 472.8)

Value are expressed as median (interquartile range) and compared using the Mann-Whitney *U *test; CA, carbohydrate antigen; * p < 0.05; ^§ ^p < 0.01; ^# ^p < 0.001

### Prognostic significance of CAC in patients with primary pancreatic cancer

Patients were followed for a median duration of 19.4 months. A total of 33 (45%) patients died of their disease during the follow-up period and 7 (9%) patients were lost to follow-up. These patients were censored at the date they were lost to follow-up.

To investigate general clinical and pathologic variables that are associated with survival after resection for pancreatic cancer, we initially performed univariate analyses (Table 4). These analyses revealed poor tumor differentiation (p = 0.02; log-rank test) to be associated with an unfavorable prognosis, whereas the association of positive lymph node status (p = 0.13; log-rank test) and R1 resection status (p = 0.08; log-rank test) with survival failed to reach statistical significance. On exploratory, univariate analyses none of the individual CAC was associated with patients' prognosis.

**Table 4:** Association of clinicopathologic variables and circulating angiogenic cytokines with cancer-specific survival: univariate analyses

	n (%)	Cancer-specific survival n (%)	*P *Value*
**Total**	74 (100)	-	-
**Gender**			0.72
Male	37 (50.0)	19 (51.3)	
Female	37 (50.0)	14 (37.8)	
**Age [years]**			0.22
< 65	30 (40.5)	14 (46.7)	
≥ 65	44 (59.5)	19 (43.2)	
**Location of the tumor**			0.74
Pancreatic head	54 (73.0)	25 (46.3)	
Pancreatic body	14 (18.9)	4 (28.6)	
Pancreatic tail	6 (8.1)	4 (66.7)	
**CA 19-9 level**			0.38
< 37 [μg/l]	20 (27.4)	8 (40.0)	
≥ 37 [μg/l]	53 (72.6)	25 (47.2)	
**CEA level**			0.59
< 2.5 [μg/l]	33 (45.2)	14 (42.4)	
≥ 2.5 [μg/l]	40 (54.8)	19 (47.5)	
**Lymph node status**			0.13
N0	54 (73.0)	26 (48.1)	
N1	20 (27.0)	7 (35.0)	
**Tumor differentiation**			0.02
Moderate (G2)	48 (68.6)	20 (41.7)	
Poor (G3)	22 (31.4)	12 (54.5)	
**Resection margin status**			0.08
R0	27 (36.5)	8 (29.6)	
R1	47 (63.5)	25 (53.2)	
**VEGF**			0.19
≥ 32.1	37 (50.0)	14 (37.8)	
< 32.1	37 (50.0)	19 (51.3)	
**VEGFR-1**			0.51
≥ 78.5	37 (50.0)	15 (40.5)	
< 78.5	37 (50.0)	18 (48.6)	
**PlGF**			0.84
≥ 11.4	37 (50.0)	18 (48.6)	
< 11.4	37 (50.0)	15 (40.5)	
**PDGF-AA**			0.53
≥ 2336.3	37 (50.0)	19 (51.3)	
< 2336.3	37 (50.0)	14 (37.8)	
**PDGF-BB**			0.10
≥ 7624.3	37 (50.0)	13 (35.1)	
< 7624.3	37 (50.0)	20 (54.1)	
**Ang-1**			0.53
≥ 29202.1	37 (50.0)	15 (40.5)	
< 29202.1	37 (50.0)	18 (48.7)	
**EGF**			0.26
≥ 234.2	37 (50.0)	17 (45.9)	
< 234.2	37 (50.0)	16 (43.2)	

* log-rank test; CEA, Carcinoembryonic antigen; CA, carbohydrate antigen.

**Figure 2:**
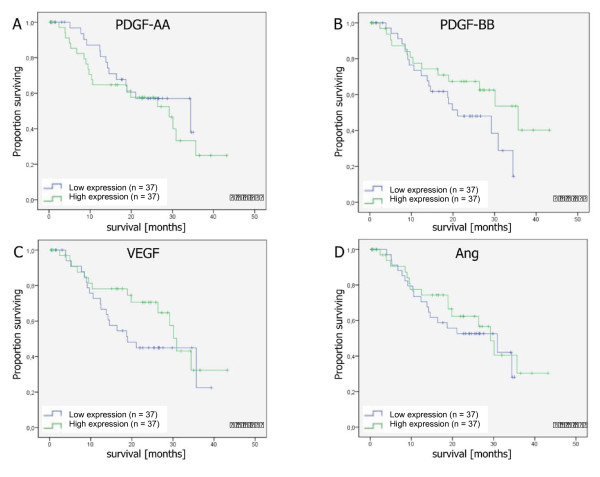
**Kaplan-Meier plots of circulating angiogenic cytokines that had significant associations with cancer-specific survival on multivariate analyses**. P-values refer to the results of univariate analyses (log-rank test).

We next constructed a multivariate Cox proportional hazards model to assess the prognostic value of our panel of CAC in patients undergoing resection for pancreatic cancer. Using the median values to dichotomize single CAC, this model included the entire panel of CAC together with clinicopathologic variables that had a prognostic value on univariate analyses. The results of this analysis revealed high levels of circulating PDGF-AA as adverse prognostic factor with respect to cancer-specific survival (hazard ratio 4.58; 95% confidence interval 1.43 - 14.69; p = 0.01). Furthermore, this analysis demonstrated high circulating levels of VEGF (HR 0.24; 95% CI 0.09 - 0.57; p = 0.002), PDGF-BB (HR 0.15; 95% CI 0.15 - 0.88; p = 0.02) and Ang-1 (HR 0.30; 95% CI 0.10 - 0.93; p = 0.04) of being associated with a favorable prognosis (Table 5). Figure 2 shows the Kaplan-Meier plots of CAC that had significant associations with cancer-specific survival on multivariate analyses.

**Table 5:** Multivariate analysis of factors associated with cancer-specific survival

Variable	Category	Hazard ratio	95% confidence interval	P value*
**Resection margin status**	R1 vs. R0	1.86	0.69 - 5.04	0.22
**Lymph node status**	Positive vs. negative	2.09	0.68 - 6.46	0.19
**Tumor differentiation**	Poor (G3) vs. moderate (G2)	2.27	0.97 - 5.32	0.06
**VEGF **[pg/ml]	≥ 32.1 vs. < 32.1	0.24	0.09 - 0.57	0.002
**VEGFR-1 **[pg/ml]	≥ 78.5 vs. < 78.5	1.52	0.63 - 3.64	0.35
**PlGF **[pg/ml]	≥ 11.4 vs. < 11.4	0.87	0.35 - 2.12	0.76
**PDGF-AA **[pg/ml]	≥ 2336.3 vs. < 2336.3	4.58	1.43 - 14.69	0.01
**PDGF-BB **[pg/ml]	≥ 7624.3 vs. < 7624.3	0.36	0.15 - 0.88	0.02
**Ang-1 **[pg/ml]	≥ 29202.1 vs. < 29202.1	0.30	0.10 - 0.93	0.04
**EGF **[pg/ml]	≥ 234.2 vs. < 234.2	1.92	0.81 - 4.54	0.14

*Cox proportional hazards regression model

## Discussion

The present study provides a comprehensive analysis of the expression and prognostic associations of CAC in patients with pancreatic cancer. Including a panel of seven CAC, the results show that certain CAC such as VEGF, PDGF-AA, Ang-1 and EGF are differentially expressed in patients with pancreatic cancer compared to healthy subjects. Furthermore, we found correlations among CAC in these patients, in particular to PDGF-AA and Ang-1. Except for PlGF the levels of all CAC, moreover, correlated with the presence of lymph node metastases, whereas there were very little associations with other clinicopathologic variables. Although the expression of individual CAC did not correlate with patients' survival on univariate analyses, the multivariate model including all angiogenic factors revealed that serum levels of VEGF, PDGF-AA, PDGF-BB and Ang-1 correlate with patients' prognosis, if the whole panel of seven CAC is considered.

The formation of new vasculature is a complex process that is mediated by a variety of cytokines [6]. Although continuous research on the molecular basis of tumor angiogenesis in solid human malignancies revealed various factors involved in this process, little is known about the interplay of these factors, in particular with respect to pancreatic cancer. The majority of experimental as well as translational studies have investigated expression and biological function of a single or very few angiogenic molecules. Our analyses, however, show multiple correlations between the different angiogenic cytokines and thus suggest that their biological functions in vivo should not be considered independently of each other, as should not be their utility in clinical practice. This notion is supported by the finding that none of the analyzed CAC correlated with patients' prognosis on univariate analyses, whereas four of these factors were significantly associated with survival on multivariate analysis including all angiogenic cytokines together with known clinicopathologic prognostic factors.

Owing to the fundamental role of angiogenesis for the growth and metastatic progression of tumors, angiogenic cytokines have been proposed as targets for systemic therapy. Furthermore, they may serve as biomarkers to predict the response or resistance to chemotherapy or anti-angiogenic therapy [28,29]. To date, however, there is no validated biological marker to accurately select cancer patients for systemic therapy [28]. One should consider that the majority of available studies did not investigate a panel of markers. Together with these data our findings suggest that the lack of one single predictive biomarker may be due to the complex interaction and involvement of various factors and due to a strong biologic and prognostic correlation between these factors. In a recently published study, Kopetz et al. examined changes of various circulating cytokines in 43 patients receiving anti-angiogenic therapy with bevacizumab for metastatic colorectal cancer and found several of these factors to be increased prior to radiological development of progressive disease [30]. While these data also indicate that the assessment of multiple factors provides more accurate information, further studies are required to validate these findings and to confirm them in other types of malignancies.

Although the lack of studies assessing multiple angiogenic factors holds true for most solid tumors, it might be of particular interest in pancreatic cancer owing to the controversial role of angiogenesis in this disease. Despite its aggressive behavior and the known overexpression of angiogenic factors, pancreatic cancer is not a strongly vascularized tumor. In line with previous studies [12-14] our data demonstrated VEGF, a key regulator of tumor angiogenesis [31], to be overexpressed in patients with pancreatic cancer. The exact biological role of VEGF and its interplay with other angiogenic cytokines in pancreatic cancer remains poorly understood and the value of VEGF as predictor of outcome in patients with pancreatic cancer is controversial [12,14,32-36]. Our finding of an inverse correlation of circulating VEGF levels with outcome is contradictory to studies that reported a positive correlation of VEGF levels with patients' survival and may in part be explained by differences in methodology. Some of these studies included patients with overt metastases, did not confirm this finding by multivariate analysis and examined expression of VEGF in tissue samples. Most importantly, the prognostic value of VEGF was commonly assessed independently of other CAC. Our data, however, indicate an inverse prognostic value of circulating VEGF in case further angiogenic cytokines are considered and, moreover, suggest that in pancreatic cancer angiogenesis is driven less strongly by VEGF but by alternative cytokines. This hypothesis is further supported by the results of clinical trials in patients with this disease that demonstrated moderate to low activity [37,38] and failed to demonstrate a survival benefit of anti-VEGF therapy [15].

Our analyses back the role of PDGFs in the molecular biology of angiogenesis in pancreatic cancer. The PDGF signaling pathway includes four ligands (PDGF-A, -B, -C, -D) and two receptors (PDGFR-α and -ß) that act as dimers and are involved in various physiological functions in embryonic development and wound healing [39]. While experimental data show PDGF signaling also to be involved in tumor growth, angiogenesis and metastasis of various cancers including pancreatic cancer [40-43], there is limited data on its biological relevance in humans. We here show that circulating PDGF-AA correlates with circulating levels of several angiogenic factors and high levels of circulating PDGF-AA are associated with poor survival in patients with pancreatic cancer and thus confirm data showing that high PDGF-AA mRNA expression levels in pancreatic tumors are correlated to shorted survival [36]. While PDGF-AA expression has already been shown to correlate with poor prognosis in other tumor entities [44,45], we also found an inverse correlation of circulating PDGF-BB levels with patients' survival. This finding is in line with experimental studies that demonstrated PDGF-BB overexpression to inhibit tumor growth in pancreatic and colorectal tumor models by increasing the pericyte content of the tumor microenvironment [46,47]. The opposed effects of PDGF-AA and PDGF-BB on tumor progression in pancreatic cancer is also supported by our finding that these cytokines are up- and down-regulated in patients with metastatic disease compared to patients with primary pancreatic cancer.

Ang-1 and Ang-2 are the two major ligands of the endothelial cell-specific tyrosine kinase receptor Tie-2. It is generally accepted that Ang-1 and 2 have important functions in the development and stabilization of vasculature [48,49], though their exact role in tumor angiogenesis and progression remains incompletely understood [50,51]. This may in part may be explained by the complexity of agonistic and antagonistic ligands for the same receptor that, moreover, act in concert with each other and other mediators such as VEGF [52-54]. There is, however, increasing experimental data demonstrating that overexpression of Ang-1 inhibits neovascularization and tumor growth in various tumor models by promoting vascular stabilization and maturation [55-58]. In line with these data, clinical studies on patients with colorectal and pancreatic cancer revealed decreased tissue expression of Ang-1 and increased expression of Ang-2 [59,60], which was, moreover, revealed as an adverse prognostic marker in colorectal cancer [61,62]. While there is limited data on the biological role of Angiopoietins in pancreatic cancer, our results also demonstrated decreased levels of circulating Ang-1 in patients with this disease. Furthermore, we show for the first time that Ang-1 levels correlate inversely with patients' prognosis and thus confirm the above preclinical data that Ang-1 inhibits tumor progression. Owing to its proposed involvement in vascular normalization by anti-angiogenic therapy [63] our data support the notion of Ang-1 as predictive biomarker of response which should be the subject of future studies.

## Conclusions

The present study analyzed the expression and biological relevance of seven CAC in patients with pancreatic cancer. The results indicate that the serum levels of certain angiogenic cytokines correlate with patients' prognosis after resection for pancreatic cancer, if a panel of several CAC is considered simultaneously. Based on these data future studies on the bioloigical role of CAC should not be limited to single molecules. The inverse correlation of certain CAC with survival may indicate why anti-angiogenic therapy has failed in patients with this disease. Furthermore, the prognostic relevance of several CAC within the analyzed panel may support the notion of multi-targeted anti-angiogenic therapy in patients with pancreatic cancer.

## Competing interests

The authors declare that they have no competing interests.

## Authors' contributions

NNR, TS, CF and MK contributed to the study design. Data acquisition was carried out by NNR, TS, CF, MH and UB. NNR, TS, UH, JW and MK contributed to data analysis. NNR and MK drafted the manuscript and TS, CF, UH MH, UB, MWB and JW revised the manuscript. Funding was obtained by MWB, JW and MK.

All authors read and approved the final version of the manuscript.

## Pre-publication history

The pre-publication history for this paper can be accessed here:

http://www.biomedcentral.com/1471-2407/11/286/prepub

## Supplementary Material

Additional file 1 **Applied adjuvant chemotherapy protocols**. Details on the adjuvant chemotherapy protocols of the study cohort. Click here for file

## References

[B1] Jemal A Siegel R Xu J Ward E Cancer statistics, 2010 CA: A Cancer Journal for Clinicians 2010 60 5 277 300 10.3322/caac.20073 20610543

[B2] Neoptolemos JP Dunn JA Stocken DD Almond J Link K Beger H Bassi C Falconi M Pederzoli P Dervenis C Adjuvant chemoradiotherapy and chemotherapy in resectable pancreatic cancer: a randomised controlled trial Lancet 2001 358 9293 1576 1585 10.1016/S0140-6736(01)06651-X 11716884

[B3] Neoptolemos JP Stocken DD Friess H Bassi C Dunn JA Hickey H Beger H Fernandez-Cruz L Dervenis C Lacaine Fo A randomized trial of chemoradiotherapy and chemotherapy after resection of pancreatic cancer The New England Journal of Medicine 2004 350 12 1200 1210 10.1056/NEJMoa032295 15028824

[B4] Oettle H Post S Neuhaus P Gellert K Langrehr J Ridwelski K Schramm H Fahlke J Zuelke C Burkart C Adjuvant chemotherapy with gemcitabine vs observation in patients undergoing curative-intent resection of pancreatic cancer: a randomized controlled trial JAMA: The Journal of the American Medical Association 2007 297 3 267 277 10.1001/jama.297.3.267 17227978

[B5] Folkman J Tumor angiogenesis: therapeutic implications The New England Journal of Medicine 1971 285 21 1182 1186 10.1056/NEJM197111182852108 4938153

[B6] Carmeliet P Angiogenesis in life, disease and medicine Nature 2005 438 7070 932 936 10.1038/nature04478 16355210

[B7] Hurwitz H Fehrenbacher L Novotny W Cartwright T Hainsworth J Heim W Berlin J Baron A Griffing S Holmgren E Bevacizumab plus irinotecan, fluorouracil, and leucovorin for metastatic colorectal cancer The New England Journal of Medicine 2004 350 23 2335 2342 10.1056/NEJMoa032691 15175435

[B8] Sandler A Gray R Perry MC Brahmer J Schiller JH Dowlati A Lilenbaum R Johnson DH Paclitaxel-carboplatin alone or with bevacizumab for non-small-cell lung cancer The New England Journal of Medicine 2006 355 24 2542 2550 10.1056/NEJMoa061884 17167137

[B9] Wong HH Lemoine NR Pancreatic cancer: molecular pathogenesis and new therapeutic targets Nature Reviews Gastroenterology & Hepatology 2009 6 7 412 422 10.1038/nrgastro.2009.89 19506583 PMC2882232

[B10] Korc M Pathways for aberrant angiogenesis in pancreatic cancer Molecular Cancer 2003 2 8 8 10.1186/1476-4598-2-8 12556241 PMC149422

[B11] Mahadevan D Von Hoff DD Tumor-stroma interactions in pancreatic ductal adenocarcinoma Molecular Cancer Therapeutics 2007 6 4 1186 1197 10.1158/1535-7163.MCT-06-0686 17406031

[B12] Ikeda N Adachi M Taki T Huang C Hashida H Takabayashi A Sho M Nakajima Y Kanehiro H Hisanaga M Prognostic significance of angiogenesis in human pancreatic cancer British Journal of Cancer 1999 79 9-10 1553 1563 10188906 10.1038/sj.bjc.6690248 PMC2362700

[B13] Itakura J Ishiwata T Shen B Kornmann M Korc M Concomitant over-expression of vascular endothelial growth factor and its receptors in pancreatic cancer International Journal of Cancer Journal International Du Cancer 2000 85 1 27 34 10585578 10.1002/(sici)1097-0215(20000101)85:1<27::aid-ijc5>3.0.co;2-8

[B14] Fujimoto K Hosotani R Wada M Lee JU Koshiba T Miyamoto Y Tsuji S Nakajima S Doi R Imamura M Expression of two angiogenic factors, vascular endothelial growth factor and platelet-derived endothelial cell growth factor in human pancreatic cancer, and its relationship to angiogenesis European Journal of Cancer (Oxford, England: 1990) 1998 34 9 1439 1447 10.1016/s0959-8049(98)00069-0 9849429

[B15] Kindler HL Niedzwiecki D Hollis D Sutherland S Schrag D Hurwitz H Innocenti F Mulcahy MF O'Reilly E Wozniak TF Gemcitabine Plus Bevacizumab Compared With Gemcitabine Plus Placebo in Patients With Advanced Pancreatic Cancer: Phase III Trial of the Cancer and Leukemia Group B (CALGB 80303) Journal of Clinical Oncology: Official Journal of the American Society of Clinical Oncology 2010 28 22 3617 3622 10.1200/JCO.2010.28.1386 PMC2917317 20606091

[B16] Philip PA Benedetti J Corless CL Wong R O'Reilly EM Flynn PJ Rowland KM Atkins JN Mirtsching BC Rivkin SE Phase III study comparing gemcitabine plus cetuximab versus gemcitabine in patients with advanced pancreatic adenocarcinoma: Southwest Oncology Group-directed intergroup trial S0205 J Clin Oncol 2010 28 22 3605 3610 10.1200/JCO.2009.25.7550 20606093 PMC2917315

[B17] Hanahan D Weinberg RA The hallmarks of cancer Cell 2000 100 1 57 70 10.1016/S0092-8674(00)81683-9 10647931

[B18] Sund M Zeisberg M Kalluri R Endogenous stimulators and inhibitors of angiogenesis in gastrointestinal cancers: basic science to clinical application Gastroenterology 2005 129 6 2076 2091 10.1053/j.gastro.2005.06.023 16344073

[B19] Ugurel S Rappl G Tilgen W Reinhold U Increased serum concentration of angiogenic factors in malignant melanoma patients correlates with tumor progression and survival Journal of Clinical Oncology: Official Journal of the American Society of Clinical Oncology 2001 19 2 577 583 10.1200/JCO.2001.19.2.577 11208853

[B20] Kwon KA Kim SH Oh SY Lee S Han JY Kim KH Goh RY Choi HJ Park KJ Roh MS Clinical significance of preoperative serum vascular endothelial growth factor, interleukin-6, and C-reactive protein level in colorectal cancer BMC Cancer 2010 10 203 203 10.1186/1471-2407-10-203 20465852 PMC2886042

[B21] Tsushima H Ito N Tamura S Matsuda Y Inada M Yabuuchi I Imai Y Nagashima R Misawa H Takeda H Circulating transforming growth factor beta 1 as a predictor of liver metastasis after resection in colorectal cancer Clinical Cancer Research: An Official Journal of the American Association for Cancer Research 2001 7 5 1258 1262 11350892

[B22] Rahbari NN Reissfelder C Muhlbayer M Weidmann K Kahlert C Buchler MW Weitz J Koch M Correlation of Circulating Angiogenic Factors with Circulating Tumor Cells and Disease Recurrence in Patients Undergoing Curative Resection for Colorectal Liver Metastases Annals of Surgical Oncology 2011 10.1245/s10434-011-1761-9 21598056

[B23] Poon RT Fan ST Wong J Clinical implications of circulating angiogenic factors in cancer patients Journal of Clinical Oncology: Official Journal of the American Society of Clinical Oncology 2001 19 4 1207 1225 10.1200/JCO.2001.19.4.1207 11181687

[B24] Poon RT-P Lau CP-Y Cheung ST Yu WC Fan ST Quantitative correlation of serum levels and tumor expression of vascular endothelial growth factor in patients with hepatocellular carcinoma Cancer Research 2003 63 12 3121 3126 12810638

[B25] Kerbel RS Tumor angiogenesis The New England Journal of Medicine 2008 358 19 2039 2049 10.1056/NEJMra0706596 18463380 PMC4542009

[B26] Rahbari NN Mollberg N Koch M Neoptolemos JP Weitz Jr Büchler MW Surgical resection for pancreatic cancer Pancreatic Cancer 2010 Springer, Berlin 971 976

[B27] Esposito I Kleeff Jr Bergmann F Reiser C Herpel E Friess H Schirmacher P Büchler MW Most pancreatic cancer resections are R1 resections Annals of Surgical Oncology 2008 15 6 1651 1660 10.1245/s10434-008-9839-8 18351300

[B28] Jain RK Duda DG Willett CG Sahani DV Zhu AX Loeffler JS Batchelor TT Sorensen AG Biomarkers of response and resistance to antiangiogenic therapy Nature Reviews Clinical Oncology 2009 6 6 327 338 10.1038/nrclinonc.2009.63 19483739 PMC3057433

[B29] Ferrara N Kerbel RS Angiogenesis as a therapeutic target Nature 2005 438 7070 967 974 10.1038/nature04483 16355214

[B30] Kopetz S Hoff PM Morris JS Wolff RA Eng C Glover KY Adinin R Overman MJ Valero V Wen S Phase II trial of infusional fluorouracil, irinotecan, and bevacizumab for metastatic colorectal cancer: efficacy and circulating angiogenic biomarkers associated with therapeutic resistance Journal of Clinical Oncology: Official Journal of the American Society of Clinical Oncology 2010 28 3 453 459 10.1200/JCO.2009.24.8252 PMC2815707 20008624

[B31] Hicklin DJ Ellis LM Role of the vascular endothelial growth factor pathway in tumor growth and angiogenesis Journal of Clinical Oncology: Official Journal of the American Society of Clinical Oncology 2005 23 5 1011 1027 10.1200/JCO.2005.06.081 15585754

[B32] Chung GG Yoon HH Zerkowski MP Ghosh S Thomas L Harigopal M Charette LA Salem RR Camp RL Rimm DL Vascular endothelial growth factor, FLT-1, and FLK-1 analysis in a pancreatic cancer tissue microarray Cancer 2006 106 8 1677 1684 10.1002/cncr.21783 16532435

[B33] Seo Y Baba H Fukuda T Takashima M Sugimachi K High expression of vascular endothelial growth factor is associated with liver metastasis and a poor prognosis for patients with ductal pancreatic adenocarcinoma Cancer 2000 88 10 2239 2245 10.1002/(SICI)1097-0142(20000515)88:10<2239::AID-CNCR6>3.0.CO;2-V 10820344

[B34] Niedergethmann M Hildenbrand R Wostbrock B Hartel M Sturm W Jr Richter A Post S High expression of vascular endothelial growth factor predicts early recurrence and poor prognosis after curative resection for ductal adenocarcinoma of the pancreas Pancreas 2002 25 2 122 129 10.1097/00006676-200208000-00002 12142733

[B35] Chang YT Chang MC Wei SC Tien YW Hsu C Liang PC Tsao PN Jan IS Wong JM Serum vascular endothelial growth factor/soluble vascular endothelial growth factor receptor 1 ratio is an independent prognostic marker in pancreatic cancer Pancreas 2008 37 2 145 150 10.1097/MPA.0b013e318164548a 18665074

[B36] Hoffmann AC Mori R Vallbohmer D Brabender J Klein E Drebber U Baldus SE Cooc J Azuma M Metzger R High expression of HIF1a is a predictor of clinical outcome in patients with pancreatic ductal adenocarcinomas and correlated to PDGFA, VEGF, and bFGF Neoplasia (New York, NY) 2008 10 7 674 679 10.1593/neo.08292 PMC2435004 18592007

[B37] Ko AH Dito E Schillinger B Venook AP Xu Z Bergsland EK Wong D Scott J Hwang J Tempero MA A phase II study evaluating bevacizumab in combination with fixed-dose rate gemcitabine and low-dose cisplatin for metastatic pancreatic cancer: is an anti-VEGF strategy still applicable? Investigational New Drugs 2008 26 5 463 471 10.1007/s10637-008-9127-2 18379729

[B38] Crane CH Winter K Regine WF Safran H Rich TA Curran W Wolff RA Willett CG Phase II study of bevacizumab with concurrent capecitabine and radiation followed by maintenance gemcitabine and bevacizumab for locally advanced pancreatic cancer: Radiation Therapy Oncology Group RTOG 0411 Journal of Clinical Oncology: Official Journal of the American Society of Clinical Oncology 2009 27 25 4096 4102 10.1200/JCO.2009.21.8529 PMC2734421 19636002

[B39] Andrae J Gallini R Betsholtz C Role of platelet-derived growth factors in physiology and medicine Genes & Development 2008 22 10 1276 1312 10.1101/gad.1653708 18483217 PMC2732412

[B40] Singh PK Wen Y Swanson BJ Shanmugam K Kazlauskas A Cerny RL Gendler SJ Hollingsworth MA Platelet-derived growth factor receptor beta-mediated phosphorylation of MUC1 enhances invasiveness in pancreatic adenocarcinoma cells Cancer Research 2007 67 11 5201 5210 10.1158/0008-5472.CAN-06-4647 17545600

[B41] Shen J Vil MD Zhang H Tonra JR Rong LL Damoci C Prewett M Deevi DS Kearney J Surguladze D An antibody directed against PDGF receptor beta enhances the antitumor and the anti-angiogenic activities of an anti-VEGF receptor 2 antibody Biochemical and Biophysical Research Communications 2007 357 4 1142 1147 10.1016/j.bbrc.2007.04.075 17462601

[B42] Kitadai Y Sasaki T Kuwai T Nakamura T Bucana CD Hamilton SR Fidler IJ Expression of activated platelet-derived growth factor receptor in stromal cells of human colon carcinomas is associated with metastatic potential International Journal of Cancer Journal International Du Cancer 2006 119 11 2567 2574 16988946 10.1002/ijc.22229

[B43] Hwang RF Yokoi K Bucana CD Tsan R Killion JJ Evans DB Fidler IJ Inhibition of platelet-derived growth factor receptor phosphorylation by STI571 (Gleevec) reduces growth and metastasis of human pancreatic carcinoma in an orthotopic nude mouse model Clinical Cancer Research: An Official Journal of the American Association for Cancer Research 2003 9 17 6534 6544 14695158

[B44] Katano M Nakamura M Fujimoto K Miyazaki K Morisaki T Prognostic value of platelet-derived growth factor-A (PDGF-A) in gastric carcinoma Annals of Surgery 1998 227 3 365 371 10.1097/00000658-199803000-00008 9527059 PMC1191274

[B45] Sulzbacher I Birner P Trieb K Träxler M Lang S Chott A Expression of platelet-derived growth factor-AA is associated with tumor progression in osteosarcoma Modern Pathology: An Official Journal of the United States and Canadian Academy of Pathology, Inc 2003 16 1 66 71 10.1097/01.MP.0000043522.76788.0A 12527715

[B46] McCarty MF Somcio RJ Stoeltzing O Wey J Fan F Liu W Bucana C Ellis LM Overexpression of PDGF-BB decreases colorectal and pancreatic cancer growth by increasing tumor pericyte content The Journal of Clinical Investigation 2007 117 8 2114 2122 10.1172/JCI31334 17641778 PMC1913488

[B47] Huang H Bhat A Woodnutt G Lappe R Targeting the ANGPT-TIE2 pathway in malignancy Nature Reviews Cancer 2010 10 8 575 585 10.1038/nrc2894 20651738

[B48] Suri C McClain J Thurston G McDonald DM Zhou H Oldmixon EH Sato TN Yancopoulos GD Increased vascularization in mice overexpressing angiopoietin-1 Science (New York, NY) 1998 282 5388 468 471 10.1126/science.282.5388.468 9774272

[B49] Augustin HG Koh GY Thurston G Alitalo K Control of vascular morphogenesis and homeostasis through the angiopoietin-Tie system Nature Reviews Molecular Cell Biology 2009 10 3 165 177 10.1038/nrm2639 19234476

[B50] Shim WS Teh M Mack PO Ge R Inhibition of angiopoietin-1 expression in tumor cells by an antisense RNA approach inhibited xenograft tumor growth in immunodeficient mice International Journal of Cancer Journal International Du Cancer 2001 94 1 6 15 11668472 10.1002/ijc.1428

[B51] Shim WSN Teh M Bapna A Kim I Koh GY Mack POP Ge R Angiopoietin 1 promotes tumor angiogenesis and tumor vessel plasticity of human cervical cancer in mice Experimental Cell Research 2002 279 2 299 309 10.1006/excr.2002.5597 12243755

[B52] Asahara T Chen D Takahashi T Fujikawa K Kearney M Magner M Yancopoulos GD Isner JM Tie2 receptor ligands, angiopoietin-1 and angiopoietin-2, modulate VEGF-induced postnatal neovascularization Circulation Research 1998 83 3 233 240 9710115 10.1161/01.res.83.3.233

[B53] Yuan HT Khankin EV Karumanchi SA Parikh SM Angiopoietin 2 is a partial agonist/antagonist of Tie2 signaling in the endothelium Molecular and Cellular Biology 2009 29 8 2011 2022 10.1128/MCB.01472-08 19223473 PMC2663314

[B54] Eklund L Olsen BR Tie receptors and their angiopoietin ligands are context-dependent regulators of vascular remodeling Experimental Cell Research 2006 312 5 630 641 10.1016/j.yexcr.2005.09.002 16225862

[B55] Ahmad SA Liu W Jung YD Fan F Wilson M Reinmuth N Shaheen RM Bucana CD Ellis LM The effects of angiopoietin-1 and -2 on tumor growth and angiogenesis in human colon cancer Cancer Research 2001 61 4 1255 1259 11245414

[B56] Stoeltzing O Ahmad SA Liu W McCarty MF Parikh AA Fan F Reinmuth N Bucana CD Ellis LM Angiopoietin-1 inhibits tumour growth and ascites formation in a murine model of peritoneal carcinomatosis British Journal of Cancer 2002 87 10 1182 1187 10.1038/sj.bjc.6600598 12402160 PMC2376191

[B57] Stoeltzing O Ahmad SA Liu W McCarty MF Wey JS Parikh AA Fan F Reinmuth N Kawaguchi M Bucana CD Angiopoietin-1 inhibits vascular permeability, angiogenesis, and growth of hepatic colon cancer tumors Cancer Research 2003 63 12 3370 3377 12810673

[B58] Hawighorst T Skobe M Streit M Hong YK Velasco P Brown LF Riccardi L Lange-Asschenfeldt B Detmar M Activation of the tie2 receptor by angiopoietin-1 enhances tumor vessel maturation and impairs squamous cell carcinoma growth The American Journal of Pathology 2002 160 4 1381 1392 10.1016/S0002-9440(10)62565-5 11943723 PMC1867215

[B59] Ahmad SA Liu W Jung YD Fan F Reinmuth N Bucana CD Ellis LM Differential expression of angiopoietin-1 and angiopoietin-2 in colon carcinoma. A possible mechanism for the initiation of angiogenesis Cancer 2001 92 5 1138 1143 10.1002/1097-0142(20010901)92:5<1138::AID-CNCR1431>3.0.CO;2-L 11571726

[B60] Durkin AJ Bloomston M Yeatman TJ Gilbert-Barness E Cojita D Rosemurgy AS Zervos EE Differential expression of the Tie-2 receptor and its ligands in human pancreatic tumors Journal of the American College of Surgeons 2004 199 5 724 731 10.1016/j.jamcollsurg.2004.07.021 15501112

[B61] Ochiumi T Tanaka S Oka S Hiyama T Ito M Kitadai Y Haruma K Chayama K Clinical significance of angiopoietin-2 expression at the deepest invasive tumor site of advanced colorectal carcinoma International Journal of Oncology 2004 24 3 539 547 14767538

[B62] Chung YC Hou YC Chang CN Hseu TH Expression and prognostic significance of angiopoietin in colorectal carcinoma Journal of Surgical Oncology 2006 94 7 631 638 10.1002/jso.20423 17066421

[B63] Winkler F Kozin SV Tong RT Chae SS Booth MF Garkavtsev I Xu L Hicklin DJ Fukumura D di Tomaso E Kinetics of vascular normalization by VEGFR2 blockade governs brain tumor response to radiation: role of oxygenation, angiopoietin-1, and matrix metalloproteinases Cancer Cell 2004 6 6 553 563 15607960 10.1016/j.ccr.2004.10.011

