# Supplementary material for: Expression and prognostic value of circulating angiogenic cytokines in pancreatic cancer
Source: BMC Cancer. 2011 Jul 5;11:286. doi: 10.1186/1471-2407-11-286 (PMC3144458; doi:10.1186/1471-2407-11-286)
Supplement: Additional file 1 — Applied adjuvant chemotherapy protocols. Details on the adjuvant chemotherapy protocols of the study cohort. [file 1471-2407-11-286-S1.DOC]

**Patients with adjuvant chemotherapy:** n = 49 (66%)

**Kind of adjuvant chemotherapy:**

Gemcitabine: n = 38 (77.5%)

GEMOX (Gemcitabine + Oxaliplatin): n = 2 (4.1%)

Capri protocol, Arm A[^1^](#_ENREF_1): Cisplatin, Interferon alpha-2b, radiation, 5-Fluorouracil: n = 2 (4.1%)

Capri protocol, Arm B[^1^](#_ENREF_1): 5-Fluorouracil, folinic acid: n = 2 (4.1%)

Unknown: 5 (10.2%)

**References:**

1. Knaebel HP, Marten A, Schmidt J, et al. Phase III trial of postoperative cisplatin, interferon alpha-2b, and 5-FU combined with external radiation treatment versus 5-FU alone for patients with resected pancreatic adenocarcinoma -- CapRI: study protocol [ISRCTN62866759]. *BMC Cancer* 2005; 5:37.
